# Case report: Long term response to growth hormone in a child with Silver-Russell syndrome-like phenotype due to a novel paternally inherited IGF2 variant

**DOI:** 10.3389/fendo.2024.1364234

**Published:** 2024-03-26

**Authors:** Silvia Ventresca, Francesca Romana Lepri, Sabrina Criscuolo, Giorgia Bottaro, Antonio Novelli, Sandro Loche, Marco Cappa

**Affiliations:** ^1^ Pediatric Section, University Hospital Arcispedale Sant’Anna, University of Ferrara, Ferrara, Italy; ^2^ Endocrinology and Diabetology Unit, Pediatric University Department, Bambino Gesù Children’s Hospital, Rome, Italy; ^3^ Laboratory of Medical Genetics, Bambino Gesù Children’s Hospital, Rome, Italy; ^4^ Pediatric University Department, Bambino Gesù Children’s Hospital, Rome, Italy; ^5^ Laboratory of Medical Genetics, Translational Cytogenomics Research Unit, Bambino Gesù Children’s Hospital, Rome, Italy; ^6^ Research Area for Innovative Therapies in Endocrinopathies, Bambino Gesù Children’s Hospital, IRCCS, Rome, Italy

**Keywords:** IGF2 variant, Silver-Russel syndrome, children, growth retardation, GH therapy

## Abstract

Silver-Russell syndrome (SRS, OMIM, 180860) is a rare genetic disorder with a wide spectrum of symptoms. The most common features are intrauterine growth retardation (IUGR), poor postnatal development, macrocephaly, triangular face, prominent forehead, body asymmetry, and feeding problems. The diagnosis of SRS is based on a combination of clinical features. Up to 60% of SRS patients have chromosome 7 or 11 abnormalities, and <1% show abnormalities in IGF2 signaling pathway genes (*IGF2*, *HMGA2*, *PLAG1* and *CDKN1C*). The underlying genetic cause remains unknown in about 40% of cases (idiopathic SRS). We report a novel *IGF2* variant c.[-6-2A>G] (NM_000612) in a child with severe IUGR and clinical features of SRS and confirm the utility of targeted exome sequencing in patients with negative results to common genetic analyses. In addition, we report that long-term growth hormone treatment improves height SDS in this patient.

## Introduction

Insulin-like growth factor 2 (IGF2) plays a pivotal role in intrauterine growth and development, and is encoded by a paternally expressed gene on chromosome 11p15.5 (1, 2). Recently, paternally inherited *IGF2* loss-of-function variants have been discovered in clusters of families with a SRS-like phenotype (3–5).

SRS (SRS, OMIM, 180860) is a rare genetic disorder with a wide spectrum of symptoms. The most common features are IUGR, poor postnatal development, macrocephaly, triangular face, prominent forehead, body asymmetry, and feeding problems. The diagnosis of SRS is based on a combination of clinical features according to the Netchine-Harbison Clinical Score System (NH-CSS) (6).

Up to 60% of SRS patients have chromosome 7 or 11 abnormalities, and <1% show abnormalities in *IGF2* signalling pathway genes (*IGF2, HMGA2, PLAG1* and *CDKN1C*). Rare familial cases of SRS have been reported with mutations in a single gene (7–9). The underlying genetic cause remains unknown in about 40% of cases (idiopathic SRS). In these cases, a comprehensive search for *IGF2* variants is recommended by the, 2017 SRS consensus (6, 8, 10–13). The use of whole exome sequencing has been reportedly shown as an effective strategy to improve the diagnostic yield in individuals with growth retardation due to genetic heterogeneity (9, 10, 14, 15).

Growth hormone (GH) is an approved growth-promoting therapy for short children born small for gestational age (SGA), including children with SRS (16). Smeets et al. reported the results of long-term GH therapy in SRS (17) in a study comparing the growth response to GH treatment in 62 SRS patients and 227 short, non-syndromic individuals born SGA. They found that mean total height gain was comparable in the two groups. While SRS subjects did not reach the same adult height due to their significant height disadvantage at the beginning of GH, the effectiveness of GH treatment was comparable in SRS and non-SRS SGA subjects. All subtypes of SRS seem to benefit from GH treatment, with a tendency towards maternal uniparental disomy for chromosome 7 and idiopathic SRS having the greatest height increase.

In this study, we report a novel paternally inherited *IGF2* variant identified by a next-generation sequencing panel in an Italian boy with a clinical diagnosis of SRS. Furthermore, we report the long term response to GH treatment in our patient. Our findings expand the spectrum of disease-causing *IGF2* variants and confirm the efficacy of GH therapy in these patients.

## Methods

The proband was referred to our institute for suspected SRS at the age of 2 years. His parents were phenotypically normal, unrelated, and clinically healthy. His father and mother’s heights were 179 and 167 cm, respectively. He was born by caesarean section performed at 37 weeks gestation for severe IUGR. He was small for gestational age (SGA) (weight -2.9 SD, length -4 SD) and showed a SRS phenotype characterized by pre- and postnatal growth failure, pronounced frontal bossing, feeding difficulties, and low BMI (NH-CSS=4). Recombinant GH was started at the age of 3 years (height -3 SDS) at a dose of 0.33 mg/kg/week.

This study was carried out at the Bambino Gesù Children’s Hospital in Rome, Italy.

Written informed consent was obtained from the parents of the patient for publication of this article. The study protocol conforms to the ethical guidelines of the, 1975 Declaration of Helsinki and has been approved by the Institution’s Human Research Committee.

Genomic DNA was extracted from blood leukocytes. SNP array and Methylation-specific MLPA were performed, ruling out abnormalities of chromosome 7 and 11. Using the Illumina NextSeq550 platform, mutational analysis was carried out by the TruSight One Sequencing Panel (Illumina, San Diego, California). The BWA Enrichment application of BaseSpace (Illumina, San Diego, CA, USA) was used to call variants, and the Geneyx Analysis program (Geneyx Genomex) was used to annotate variants and prioritize potential genes based on phenotypes.

## Results

We found a heterozygous splice variant c.[-6-2A>G] (NM_000612) in the *IGF2* gene. The American College of Medical Genetics (ACMG) rated the variation as possibly pathogenic. Using established procedures, Sanger sequencing was used for segregation analysis.

**Figure 1:**
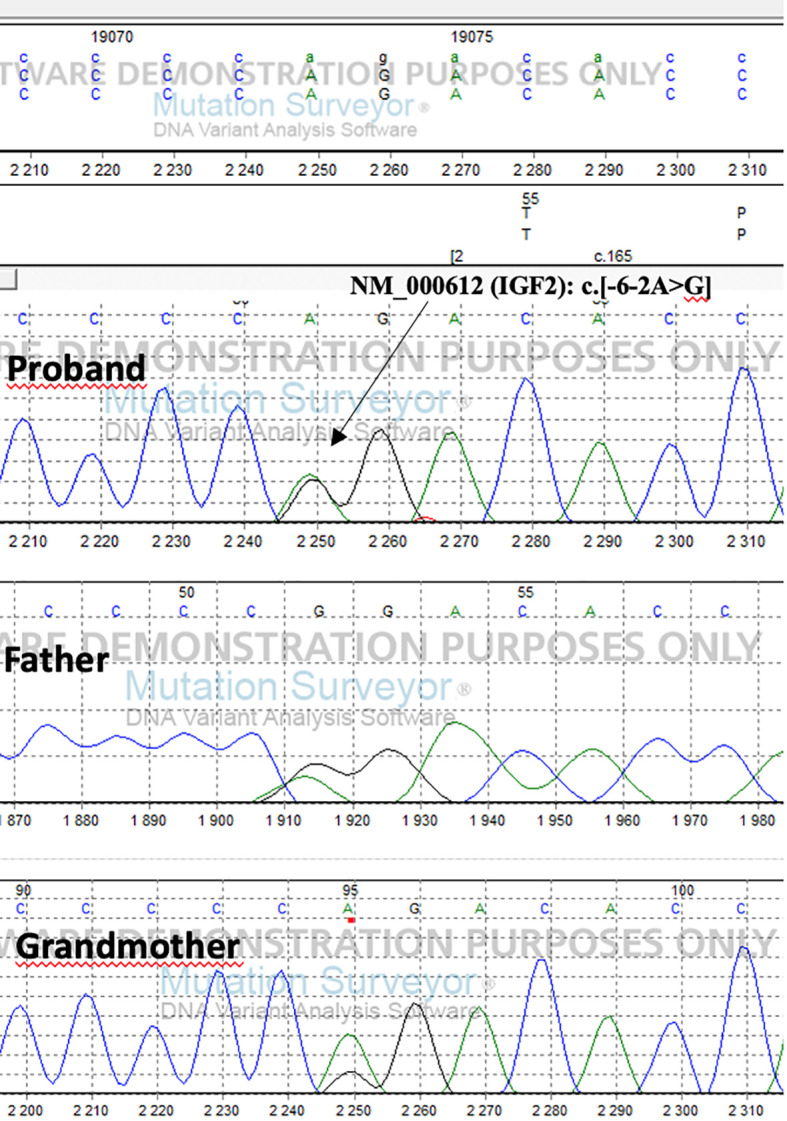
Results of sequence analysis of the proband, his father and grandmother.

The detected genetic variation segregates on the paternal side. The patient’s father inherited the mutation from his mother (**Figure 1**) Due to the maternal imprinting of the *IGF2* gene, the patient’s father is an asymptomatic carrier.

**Figure 2:**
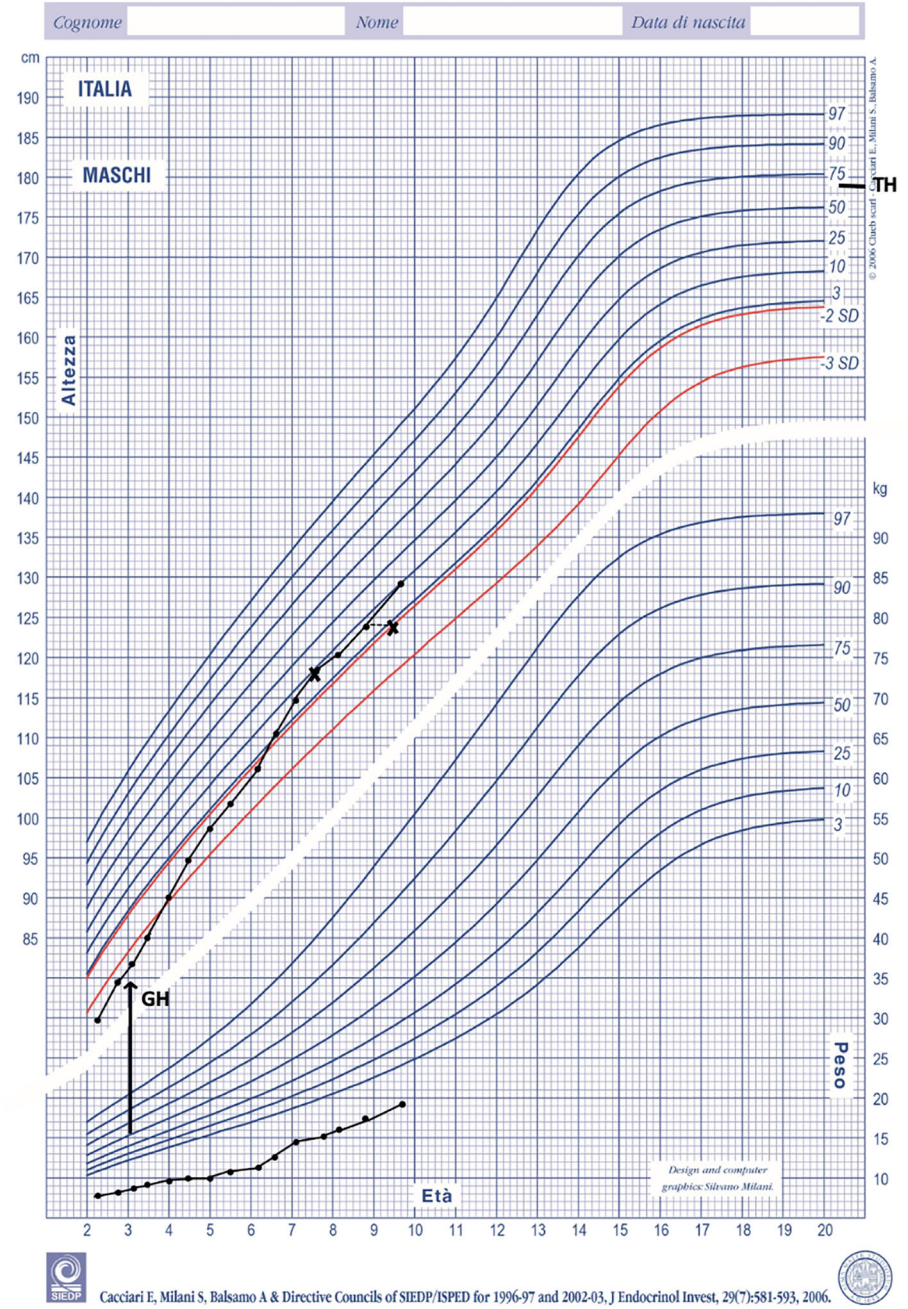
Height and weight during GH treatment plotted on Italian growth chart for boys (18). TH, target height.

GH therapy produced a positive growth response. His growth rate increased from 5 cm/y at baseline to 8 cm/y during the first year of treatment. At the age of 8 his growth rate was 7 cm/year with a bone age of 7 years. (**Figure 2**). His height SDS increased from -3.50 SDS at baseline to -1.29 SDS at the age of 8. No side effects and no serious illnesses were observed during treatment. Psychomotor development was normal.

Serum IGF1 levels were normal at baseline and increased but remained in the normal range during the first 1,5 years. Then increased above 2 SDS and remained consistently elevated throughout the treatment period (last measurement +3.17 SDS) (**Table 1**).

**Table 1:** IGF1 concentration, GH dose, growth velocity, BMI and bone age during treatment.

Age (years)	IGF1 (μg/L)	IGF1 Standard deviation Score (SDS)	rhGH dose (mg/kg/week)	Growth velocity (cm/y)	BMI Standard deviation Score (SDS)	Bone age (years)
3,5	244,4	0,06	0,33	6	-2.64	/
4,5	336,1	0,78	0,33	8,6	-2.52	/
5	325	0,72	0,24	6,5	-2.97	5,2
6,5	479	2,85	0,29	8,2	-2.90	/
7,7	522	3,59	0,27	6,3	-2.79	7,8
8,8	447	2,28	0,25	7	-2.56	9,6
9,7	320	3,17	0,23	7,2	-2.46	/

## Discussion

We identified a novel genetic abnormality (variation c.[-6-2A>G] NM_000612) in the *IGF2* gene in a young child whit pre- and postnatal growth retardation and clinical features of SRS. Exome capture was performed on the patient and parental DNA samples after exclusion of molecular abnormalities on chromosomes 7 and 11 (maternal uniparental disomy, ICR1 hypomethylation, submicroscopic imbalances, and *CDKN1C* variations) by SNP array and methylation-specific MLPA. This variant has not been observed in gnomAD, 1,000 Genomes, or Sequencing Initiative Suomi database (SISu), it has not been reported in dbSNP, ClinVar, or HGMD and is classified as possibly pathogenic by the American College of Medical Genetics. The presence of the *IGF2* variant was confirmed by Sanger sequencing, and segregates on the paternal side. Thus, it was inherited from the father, who had inherited it from his mother. There were no similar cases in the family. These findings are consistent with maternal imprinting of the *IGF2* gene.

Our results confirm that specific molecular analyses are useful to elucidate the diverse molecular spectrum of SRS and clinically related disorders. Confirming previous observations (5), whole genome sequencing, long-read sequencing (third generation sequencing) and transcriptomics allow the identification of most molecular abnormalities in a single genetic study.

Meyer et al. (10) performed genetic analysis in 75 SRS phenotypes, and identified a disease-causing variant in 21/75 patients, including variants in known SRS genes (*IGF2, PLAG1, HMGA2*). Several patients carried variants in genes not previously linked to the diagnosis of SRS. A recent clinical study in Japan (4) found five patients with clinical features of SRS and novel *IGF2* polymorphisms in the paternal allele. *IGF2* epimutations are clinically associated with SRS with various phenotypic abnormalities. This is most likely due to the different *IGF2* expression patterns in the target organs (4). The study by Yamoto et al. (5) of a family with a paternally inherited nonsense mutation in IGF2 confirmed that IGF2 variations could account for the combined phenotypes of the four SRS individuals (extrinsic finger, hypo-masculinization, developmental delay and placental hypoplasia). In addition, they observed that serum IGF1 and IGFBP3 levels were significantly higher than normal in these patients (19). In the study of Smeets et al. (17) baseline IGF1 concentrations were in the normal range in patients with SRS. However, after one year of GH treatment median IGF1concentration was above 2 SDS in the subgroup of patients with 11p15 aberrations. Consistent with these findings, our patient also had normal baseline IGF1 concentrations which increased above 2 SDS on GH treatment. IGF1 remained above 2 SDS even after reduction of the GH dose in our patient. The reasons for the elevated IGF1 concentrations are not clear. Previous studies have suggested reduced IGF1 sensitivity in SRS patients with 11p15 epimutation (20). Reduced IGF1 sensitivity may thus be speculated also for our patient.

Short children born with SGA are a recognized indication for GH therapy. Treated subjects reach a mean height gain of 1.5 SDS compared with 0.25 SDS in untreated subjects (21). The dose of GH recommended for SGA (0.22 mg/kg/week) is higher than that commonly used in children with GHD (21). Recently, beneficial effects of GH treatment on growth and body composition have also been reported in SGA children with SRS (17). Total height gain during GH treatment was similar in SGA children and in SGA children with SRS at a dose of 0.22 mg/kg/week. In addition, the growth response was not significantly different between children with different genetic subtypes. Interestingly, a correlation between age at start of therapy and long-term outcome has been recently reported in short children born SGA (22). Confirming these findings, our patient exhibited a positive response to GH treatment. Our patient’s primary diagnosis was SRS, and given the pronounced height deficit already present at the age of 3 years we asked permission to the *ad hoc* local Committee to start treatment with GH. We started GH treatment using the maximum allowed dose of 0,33 mg/Kg/week and did not adjust the dose according to IGF1 concentration, but rather to growth velocity. We have reduced the dose gradually as growth velocity reached a plateau. After 5 years of treatment, his height SDS increased from -3.50 at baseline to -1.29 with a total height gain of 1,89 SDS. Weight did not increase over height during treatment and as a result BMI was consistently low. To our knowledge there are no studies on the effect of long-term GH treatment on BMI in children with SRS. A recent report, however, speculated that lower BMI could result from differences in body composition including reduced fat mass in GH‐treated individuals (23).

We have confirmed that targeted exome sequencing to search for *IGF2* gene mutations is useful in children with IUGR and SRS-like phenotype. All *IGF2* variants associated with an SRS-like phenotype reported so far (**Table 2**), including this novel one, presented with pre- and postnatal growth restriction and feeding difficulties, and 80% had also developmental delay and/or intellectual disability. Our patient had severe prenatal growth restriction, feeding difficulties and severe growth retardation, which improved with GH treatment.

**Table 2:** Summary of the *IGF2* variants reported in patients with SRS phenotype.

Nucleotide change	Protein substitution	Reported phenotype	Birth length (SDS)	GH Rx	Ref
c.23C>A	p.S8X	IUGR	-3.0	No	3
c.-6-1G>C	affect splicing	SRS	-3.0	No	4
c.134G>C	p.C45S	SRS	-4.0	Yes	4
c.209G>A	p.C70Y	SRS	-3.0	Yes	4
c.211T>C	p.C71R	SRS	-2.5	Yes	4
c.110_117delTGGTGGACinsAGGTAA	p.(Leu37GlnfsX31)	SRS	-4.0	Yes	5
c.97T>A	p.C33S	SRS	NR	No	5
c.78C>G	p.Y26X	SRS	-3.5	Yes	8
c.158_159dupGC	p.(Arg54AlafsX7)	SRS	-5	Yes	8
c.213T>G	p.C71W	SRS	-3.5	No	10
c.157 + 5G>A	affect splicing	SRS	NR	No	11
c.157 + 3A>C	affect splicing	SRS	-2.3	No	12
c.99C>A	p.C33X	SRS	NR	Yes	13
c.101G>A	p.G34D	SRS	-3.0	Yes	14
c.122T>G	p.Leu41Arg	SRS	-3.4	No	15

GH Rx, GH therapy.

In conclusion, the use of a next-generation sequencing protocol that analyses multiple genes simultaneously allows early detection of pathogenetic mutations in various genetic conditions with high standards of coverage and quality. This can lead to early diagnosis, especially in patients with mild or atypical features, and more appropriate genetic counselling and clinical management, including the possibility of early initiation of GH therapy.

## Data availability statement

The datasets presented in this article are not readily available because of ethical and privacy restrictions. Requests to access the datasets should be directed to the corresponding author/s.

## Ethics statement

The studies involving humans were approved by the Ethics Committee of Bambino Gesù Children Hospital of Rome. The studies were conducted in accordance with the local legislation and institutional requirements. Written informed consent for participation in this study was provided by the participants’ legal guardians/next of kin. Written informed consent was obtained from the individual(s), and minor(s)’ legal guardian/next of kin, for the publication of any potentially identifiable images or data included in this article.

## Author contributions

SV: Writing – original draft, Writing – review & editing. FL: Writing – original draft, Writing – review & editing. SC: Writing – original draft, Writing – review & editing. GB: Writing – original draft, Writing – review & editing. AN: Writing – original draft, Writing – review & editing. SL: Writing – original draft, Writing – review & editing. MC: Writing – original draft, Writing – review & editing.
